# Peptide Hydrogels – Versatile Matrices for 3D Cell Culture in Cancer Medicine

**DOI:** 10.3389/fonc.2015.00092

**Published:** 2015-04-20

**Authors:** Peter Worthington, Darrin J. Pochan, Sigrid A. Langhans

**Affiliations:** ^1^Nemours Center for Childhood Cancer Research, Alfred I. duPont Hospital for Children, Wilmington, DE, USA; ^2^Department of Biomedical Engineering, Delaware Biotechnology Institute, University of Delaware, Newark, DE, USA; ^3^Department of Materials Science and Engineering, Delaware Biotechnology Institute, University of Delaware, Newark, DE, USA

**Keywords:** three-dimensional cell culture, hydrogel, functionalization, cancer, matrix

## Abstract

Traditional two-dimensional (2D) cell culture systems have contributed tremendously to our understanding of cancer biology but have significant limitations in mimicking *in vivo* conditions such as the tumor microenvironment. *In vitro*, three-dimensional (3D) cell culture models represent a more accurate, intermediate platform between simplified 2D culture models and complex and expensive *in vivo* models. 3D *in vitro* models can overcome 2D *in vitro* limitations caused by the oversupply of nutrients, and unphysiological cell–cell and cell–material interactions, and allow for dynamic interactions between cells, stroma, and extracellular matrix. In addition, 3D cultures allow for the development of concentration gradients, including oxygen, metabolites, and growth factors, with chemical gradients playing an integral role in many cellular functions ranging from development to signaling in normal epithelia and cancer environments *in vivo*. Currently, the most common matrices used for 3D culture are biologically derived materials such as matrigel and collagen. However, in recent years, more defined, synthetic materials have become available as scaffolds for 3D culture with the advantage of forming well-defined, designed, tunable materials to control matrix charge, stiffness, porosity, nanostructure, degradability, and adhesion properties, in addition to other material and biological properties. One important area of synthetic materials currently available for 3D cell culture is short sequence, self-assembling peptide hydrogels. In addition to the review of recent work toward the control of material, structure, and mechanical properties, we will also discuss the biochemical functionalization of peptide hydrogels and how this functionalization, coupled with desired hydrogel material characteristics, affects tumor cell behavior in 3D culture.

## Introduction

### History of cancer models

Over the past decades, fundamental biological research and translational and clinical studies have tremendously increased our understanding of cancer, transforming it from a poorly understood and mostly deadly disease into one with ever evolving therapeutic options and increasing survival rates. While a large variety of cancer models is available, spanning from cells cultured in a monolayer (1, 2), to cells cultured within a complex substrate, to animal models (3), and, ultimately, human cancer tissues and clinical trials (4), much of our understanding of cancer has resulted from research on cultured cells utilizing various cell models (5, 6). As each model has its own set of advantages and disadvantages, the best choice often becomes a tradeoff between simplicity of setup vs. clinical transfer of results to human patients (7).

The growing range of cancer model options available today was not available at the turn of the 19th to the 20th century. One of the earliest tumor models created was designed by Carl Jensen (8), where he transplanted mouse sarcomas into healthy mice and measured tumor growth to estimate the vitality of the transplanted cancer. This type of tumor transplantation would continue as the primary animal model until the 1980s, with the creation of transgenic mice and the ability to readily develop *in vivo* cancer models with specific gene mutations (9). In addition to the early animal cancer models, stable cancer cell lines were first developed starting in the 1950s with Hela cells being the most commonly used and oldest cancer cell line available (10). These immortalized cell lines allowed for prolonged, controlled cellular studies when cultured in glass Petri dishes or, more recently, on tissue culture polystyrene. However, while monolayer cultures undoubtedly have played and still play a crucial role in cancer research, there remains a vast jump in complexity from two-dimensional (2D) cell cultures to animal models often resulting in clear differences between experimental findings and clinical reality (11).

Beginning in the early 1980s, researchers began to address the large differences between 2D cell culture and the *in vivo* environment by adding more intricacy to 2D cell culture with testing the effects of new substrate materials on cells in culture (12–14). It is now well accepted that 2D cultures can show large differences in cell phenotype by controlling the cell culture scaffold. For example, on 2D hyaluronic acid (HA) scaffolds, changing the elasticity of the matrix through crosslinking or adding collagen ligands affected the organization of the actin cytoskeleton (15). Another study showed that matrix stiffness controlled stem cell differentiation and lamin levels (16). Hydrophobicity of the scaffold was shown to control adhesion of cells to the matrix and ultimately what phenotypes the cells display (17). Additional complexity can be added by growing cells *in vitro* in three-dimensional (3D) matrices. Culturing cells within a 3D substrate is a relatively new culture method that seeks to combine the simplicity of *in vitro* cell culture with creating results more relevant to a 3D *in vivo* environment while also helping to minimize the costs and variability associated with animal models (18). This will be of particular interest in the development of new lead compounds for cancer therapy by high-throughput screening (HTS) of small molecule libraries. While HTS remains a promising step in cancer drug development, its value has been limited as prediction of the clinical success of new drug candidates proved to be difficult (19). One of the reasons for this lack of reliability to predict *in vivo* efficacy has often been ascribed to the fact that most HTS screenings are done using traditional 2D cultures of cancer cells. While 2D cultures are convenient and can easily be automated, new 3D matrices are well suited to provide more physiological and thus predictive platforms for HTS and drug discovery in cancer.

## Advantages of 3D Cell Cultures

When comparing 2D and 3D cell cultures at a cursory level, it should seem clear that 3D cell constructs are more true to *in vivo* conditions as tissues and tumors are 3D structures of extracellular matrix (ECM) and multiple cell types that interact in a complex manner rather than being a simple monolayer or a series of stacked cellular monolayers (20, 21). In a 3D environment, cells respond differently to stimuli as compared to 2D monolayers because of multiple variables in the environment surrounding the cells (22) and the material that constitutes the scaffold (e.g., protein, synthetic polymer, or a combination of the two) has a large impact through its properties such as density (23), porosity (24), and stiffness (25, 26). Chemical functionalities in 3D scaffolds can also affect cell behavior and the density of attachment ligands controls the amount of focal adhesions in a cell. While in monolayers, these focal adhesions are limited to the interactions of the basal membrane with the surface of the tissue culture dish; these interactions encompass the entire cell surface in a 3D matrix (27, 28). Such cell–matrix interactions often result in differences in cell morphology within a 3D matrix. For example, in gelatin hydrogels, cell alignment and elongation can be controlled (29).

Another significant difference between 2D and 3D environments is the availability of small molecules such as glucose, amino acids, and other growth factors that are usually added to culture medium and that of oxygen. In 2D monolayers, usually all cells have direct access to these nutrients; while in 3D cultures, the availability of small molecules depends on diffusion rates and local environments within the scaffold (30). This results in concentration gradients throughout the matrix that can more closely mimic a tissue environment with cells encapsulated further from the media having decreased exposure to additives and oxygen (31). Such chemical gradients can be responsible for various cell responses including differentiation and oxygen gradients are particularly important in tumor development (32). 3D cultures also allow for the formation of spheroids that are instrumental to many cancer cell studies including breast cancer (33). Such spheroids have been shown to have an LD50 value much closer to *in vivo* values, which may explain the lack of *in vivo* efficacy often found in drug development that relies on 2D monolayers (34).

*In vivo* cells exist in a complex aqueous 3D environment encompassing primarily proteins and polysaccharides. The cells interact with their environment by binding to these proteins thereby activating signaling pathways that crosstalk with growth factor signaling cascades to integrate environmental cues. For example, matrix signals contribute to the integrin-mediated control of mesendoderm differentiation in which specific matrix glycans govern cell fate (35). Cancer cells are adept at altering their microenvironment to promote tumor growth and metastasis. In the case of thyroid cancer harboring the BRAF mutation, the mutation promotes upregulation of genes associated with ECM remodeling, resulting in a more aggressive cancer (36, 37). Each facet of the microenvironment helps to control and direct cell fate (38, 39), and consideration of the optimal scaffold for 3D cell culture is of paramount importance as it will directly affect the phenotype and behavior of cells in culture. An exciting new application will be the modeling of tumor angiogenesis and evaluation of the angiogenic capability of tumor cells in 3D (40). While there are various matrix supports available to recreate complex culture environments due to their unique ability to recapitulate a realistic *in vivo* environment, hydrogels have been the overwhelming choice for use in 3D cell culture.

## Hydrogels as 3D Cell Culture Scaffolds

A hydrogel is a dilute polymer or supramolecular network with given structure and network properties obtained by intermolecular crosslinks in the case of a polymer molecular network or by interfibrillar crosslinks in the case of supramolecular fibrillar hydrogel networks (41, 42). A hydrogel is mostly water, usually defined as over 95% by volume, but the material displays solid-like material properties in the quiescent state (43). Hydrogels are ideal materials to use as 3D cell culture scaffold because of the similarities in material properties and, when properly designed, the similarities in biological properties to the ECM (43). A common example of a hydrogel is gelatin, a protein mixture derived from cleaving collagen that is used in many different processes; including cooking, photography, and pharmaceuticals. For use in cell culture, there are multiple types of gelatin depending on the collagen source, which have different mechanical properties (44). When comparing porcine and piscine gelatin, there are similarities due to their base collagen being alike but the different ratios of amino acids result in piscine gelatin being weaker rheologically and having a lower melting temperature (45).

For the purpose of this review, hydrogels will be categorized by their source, biologically or synthesized, and by their material, naturally occurring or by human design. Natural hydrogels are those that are found in nature, and are taken from a biological source, and include collagen, matrigel, which constitute ECM proteins and alginate that originates from cell walls of algae. Due to their natural source, they are usually biocompatible. On the other hand, they are complex and ill-defined scaffolds that are difficult to tune mechanically.

There are multiple types of synthetically produced hydrogel scaffolds including non-natural polymers like polyethylene glycol (PEG) (46) and natural polymers like the polysaccharide HA (47–49). Unnatural synthesized polymer hydrogels have the advantage that they are usually easy to tune by controlling material aspects either through synthesis or crosslinking. They are also comparatively inexpensive and the material is reproducible and shows consistent results. However, the lack of biological moieties may be a shortcoming when attempting to reproduce natural EMC but biological peptides often can be cross-linked to the scaffold to improve functionality (50, 51). Like synthetic polymers, natural synthesized polymers share advantages in regard to material tunability and batch-to-batch consistency with reproducible results (52–54). Natural synthesized hydrogels can also be highly biocompatible because of the biological nature of the base material requiring less frequently functionalization as biological moieties are part of the primary chain. Unfortunately, the material cost can be high depending on the type of synthesis required.

## Current Standard Natural Cell Culture Hydrogels

Hydrogel materials made from natural proteins, for example collagen and matrigel, are a common choice as 3D cell culture scaffolds due to their biocompatibility, adhesive properties, and inclusion of growth factors, often resulting in cellular phenotypes typically seen *in vivo*, including spheroid formation, controlled differentiation, and directed growth.

### Collagen

Collagen is a fibrillar protein made of three alpha helices coiled together into a triple helix. It is the most abundant protein in humans and provides for structure and function in the ECM, making it a logical choice as an *in vitro* cell culture scaffold (55–57). Cells attach to collagen through integrins thereby activating cell signaling pathways that control cell survival (58), cell growth, and differentiation (59). The cellular phenotype of cells grown in collagen may differ vastly from monolayer cultures. For example, Madin-Darby canine kidney (MDCK) cells grow in 2D cultures as tight monolayers but form spheroids in collagen (60). Changes in collagen nanostructure can control morphology and the osteogenic potential of cells in culture (61), while changes in collagen stiffness can alter fibroblast proliferation (58). Collagen has also been seen to improve survival outcomes for cell transplants into rats (58).

### Matrigel

Matrigel is a collection of proteins and growth factors extruded from Englebreth–Holm Swarm mouse tumors (13). It is mainly made of the proteins collagen, laminin, and enactin, but also includes multiple growth factors like basic fibroblast growth factor, epidermal growth factor, insulin like growth factor, transforming growth factor beta, platelet derived growth factor, and nerve growth factor (62). Because it is produced from excreted ECM and has been minimally processed, Matrigel is a good mimic of *in vivo* cellular conditions to study cellular phenotypes (63, 64). For example, breast cancer cells cultured on matrigel are more susceptible to drug treatment compared to monolayer cultures (11), and macrophages can be induced to express endothelial lineage markers in an angiogenic environment with matrigel (65).

While naturally derived collagen and matrigel hydrogel materials show success for many cell culture conditions, there is significant room for improvement of the materials for 3D culture. Some of the limitations of biologically derived materials result from the manufacturing process from live tissue, resulting in a complex, chemically not well-defined scaffold consisting of more than 1800 proteins (62, 66). The presence of multiple growth factors together with batch-to-batch variability of the purified scaffold may interfere with biological studies of signaling pathways or pharmacological investigations on drug-induced effects (67). To overcome this obstacle, growth factor-reduced matrigel has been developed for applications requiring a more highly defined basement membrane preparation, whereas high concentration matrigel appears to be better suited for *in vivo* applications. A study comparing the two scaffolds using patients’ degenerative disk cells found that cells grown on top of either scaffold displayed similar phenotypes, but the proliferation rate was higher for cells grown on the reduced growth factor scaffold (68). However, an ill-defined hydrogel material cannot be approved for human implantation leading to road blocks in translating basic research into clinical applications. Thus, there is clearly a need for new materials with the biological functionality of naturally occurring ECM and the capability of designing specificity in biological and material properties (69). Synthetic hydrogel materials are ideally suited to address these limitations.

## Established and Upcoming Synthetic Peptide Hydrogels

The evolving field of small peptide-based hydrogel materials allows for material definition and design applicable for future clinical biomedical efforts and provides new scaffolds for 3D cell culture to address important questions in cell biology, drug delivery/discovery, and tissue engineering. The molecules, while diverse in primary structure, have a similar, general design. The peptides tend to be amphiphilic, relying on intramolecular folding and intra- and intermolecular physical interactions, controlled by pH and salt, for gelation through intermolecular assembly. Most final hydrogel materials also have a nanofibrillar structure. One major advantage of using synthetic peptides is the ease by which alterations can be introduced into the hydrogel scaffold by amino acid substitution/addition (both natural and non-natural), extension/shortening of the peptide sequence, or functional epitope addition at the termini of peptide chains or as side chains to a peptide sequence (70–73). Thus, the peptides described in the following chapter in fact represent a family of peptides with a basic structure, and if applicable, additional functional alterations. Each section will examine different short sequence amino acid hydrogels families, their unique characteristics, advantages and disadvantages, and potential as a 3D cell culture scaffold. The peptides being presented have been around for varying amounts of time. The older more established peptides have been used to culture a variety of cell lines including cancer cells and have undergone several functional alterations to improve culture conditions. The more recently developed peptides are not as well characterized and some were originally developed as a drug delivery vehicle and are only now being discovered as suitable 3D culture scaffolds. Despite the fact that some of these hydrogels have not yet been used for the culture of cancer cells, we decided to include these peptides in this review. They should not be overlooked as they show great promise for future applications in cancer research, including modeling of the tumor microenvironment, enrichment of cancer stem cells and drug discovery. For example, while HTS remains a promising initial step in building new classes of lead compounds, and particularly in cell line models for cancer, its value is limited in predicting clinical effectiveness. One of the reasons for this lack of reliability to predict *in vivo* efficacy has often been ascribed to the fact that most HTS screenings were done using traditional 2D cultures of cancer cells. While 2D cultures are convenient and can easily be automated, compelling evidence suggests that cells cultured in non-physiological 2D conditions differ from cells grown in the more *in vivo* like 3D systems. Thus, a 3D culture model is expected to be a better platform for drug discovery and is likely more predictive of efficacy of potential drugs for future preclinical studies and clinical trials. Collagen or matrigel are commonly used 3D matrices that provide an *in vivo* like environment. However, due to their natural origin, the batch-to-batch variation is considered a major hindrance to obtain reproducible results. Natural matrices also limit the possibility of mimicking different tissue environments as they only have limited capabilities for their chemical and mechanical properties to be modified. Synthetic peptide hydrogels can overcome these limitations and can provide an optimized biologically active cell environment with tunable porosity, permeability, and mechanical stability. Synthetic matrices not only offer more defined properties than matrices obtained from natural resources but also allow for controlling of adhesive moieties (e.g., functionalization with RGDS peptides), controlled inclusion of proteolytic sites, and defined mechanical properties such as material stiffness and pore size to enhance nutrient exchange and improve cell proliferation. While HTS screening in 3D cultures is still in its infancy, it is one of the most promising applications of synthetic peptide hydrogels.

### EAK16 and RADA16

**Figure 1:**
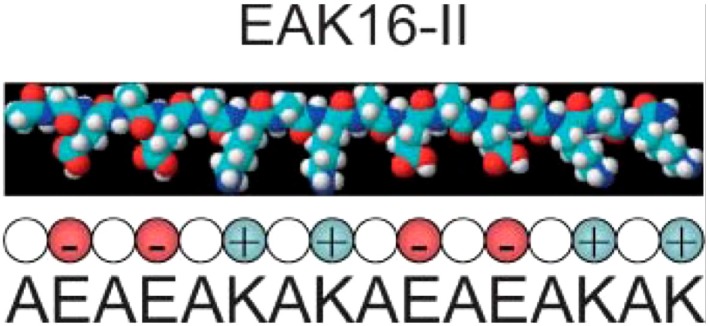
**The EAK16 sequence**. Reprinted with permission from Ref. (81). Copyright 2004 Biophysical Society.

EAK16 is an amphiphilic peptide and was one of the earliest peptide hydrogels used for cell culture (74). Its amino acid sequence, AEAEAKAKAEAEAKAK (Figure 1), was discovered in yeast (75), and subsequently a second peptide designated RADA16 was developed based on reproduction of the molecular interactions that cause EAK16 to gel. These gels initially were utilized as a substrate for 2D cultures and have been used for quite a variety of cell types including HIT-T15, CEF, HFF, HepG2, MG63, Hela, HEK293, 3T3, PC12, and SH-SY5Y cells (76). A typical protocol for a culture setup includes mixing of the peptide solution with culture medium and allowing the mixture to dry overnight. Plating the cells on top of the substrate enables standard cell culture procedures, proliferation assays, and fluorescence microscopy (76). EAK16 was subsequently also developed into a true 3D culture model, where a cell/sucrose suspension was mixed directly with the peptide solution creating a cell encapsulated gel (77). RADA16 was used for a 3D ovarian cancer model to study cell invasion and drug resistance. When compared to 2D cultures, the RADA16 cell system showed stronger resistance to anticancer agents, curcumin and paclitaxel (73). In a different study, RADA16 was used as a vehicle to deliver Schwann cells to rat spinal cord injuries, and the RADA16 group showed the biggest improvement in mobility as measured by the Basso Beattie and Bresnahan test (78). A similar study used RADA16 functionalized with IKVAV, a sequence derived from laminin, to deliver neural stem cells to traumatic brain injury in rats. Greater cell proliferation was shown using RADA16 and the ability to direct cell differentiation fate with the functionalized matrix caused more mature neurons to form (79). While these are promising results, the major disadvantage of EAK16 and RADA16 are their mechanical properties. The stiffness is very low compared to natural tissue resulting in a lack of appropriate rheology. Groups have attempted to increase stiffness by adding functional groups with some success. A notable example was the addition of GPGGY to RADA16, an amino acid sequence inspired by a protein in spider silk (80).

### Fmoc-FF and Fmoc-RDG

**Figure 2:**
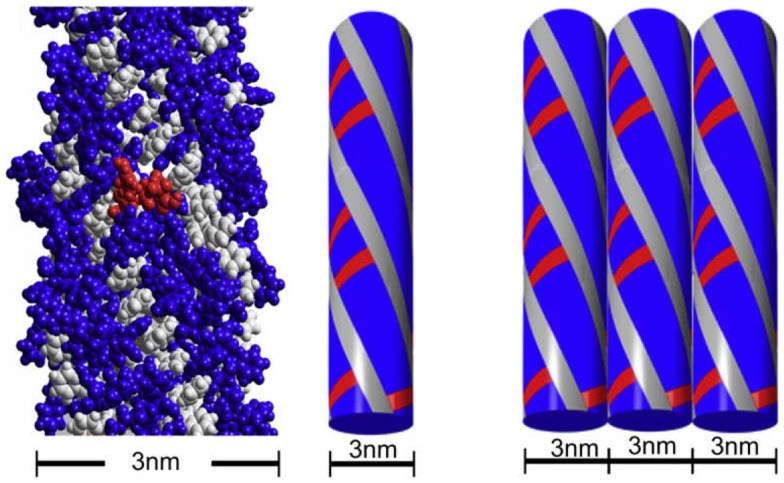
**The fibrillar structure of Fmoc-FF and Fmoc-RGD**. Reprinted with permission from Ref. (84). Copyright 2009 Elsevier Ltd.

Fluorenylmethoxycarbonyl-diphenylalanine (Fmoc-FF) and fluorenylmethoxycarbonyl arginine–glycine–aspartic acid (Fmoc-RDG) form hydrogels based on aromatic interactions (82–84) and have been successful as a scaffold for cell culture (85, 86). The hydrogel is a relatively simple gelator relying on a few peptides and Fmoc groups and is formed by pi–pi staking between the Fmoc groups, thereby forming 3 nm fibrils which interact laterally to create “flat ribbons.” RGD groups were added to increase cell attachment (Figure 2, in red) and these gels produce a viable 3D encapsulated cell culture with HDFa cells. As such, the scaffolds are suitable for assessment by various methods including fluorescence microscopy and the MTS assay (84). The cell gel constructs are formed by dissolving the peptide in DMSO followed by dilution in an aqueous solution at pH 10 (Fmoc-FF) or pH 3 (Fmoc-RGD), followed by adjustment of the pH to physiological conditions. The solutions can then be mixed with cells in culture medium (87). The gel forms quickly <1 min and has a G′ of around 780 Pa with the final stiffness of the gel being primarily dependent on the final pH (88). Within this system, addition of amino acids other than RGD were tested as well resulting in hydrogel constructs that were more suitable for some cell lines than others. For example, Fmoc-Lysine, Fmoc-Glutamic acid, or Fmoc-Serine constructs were able to grow human dermal fibroblasts but only Fmoc-Serine allowed for the growth of chondrocytes and 3T3 cells (89). Other stacking groups, naphthalene and benzyloxycarbonyl, in place of the Fmoc group have been shown to create fibrils that support chondrocyte growth (90). Changes to the sequence included different combinations of phenylalanine and RGD in order to avoid mixing of different peptides (91). Recently, work has been done to improve the biocompatibility of the gelation process by using glutathione to cleave a sulfide bond on the pregelator that would allow the peptide to gel avoiding the use of DMSO (92). Another group altered and improved gelation by halogenating the phenyl ring on phenylalanine, a scaffold that could be used to culture 3T3 cells after adding RGD to the system (72). Yet, other groups have added different amino acids to improve cell attachment and confirmed the importance of the RGD group (93). While Fmoc groups are not normally found in the ECM, these gels exhibit decent stiffness and appropriate rheology and have proven suitable as 3D cell culture scaffold.

### h9e

**Figure 3:**
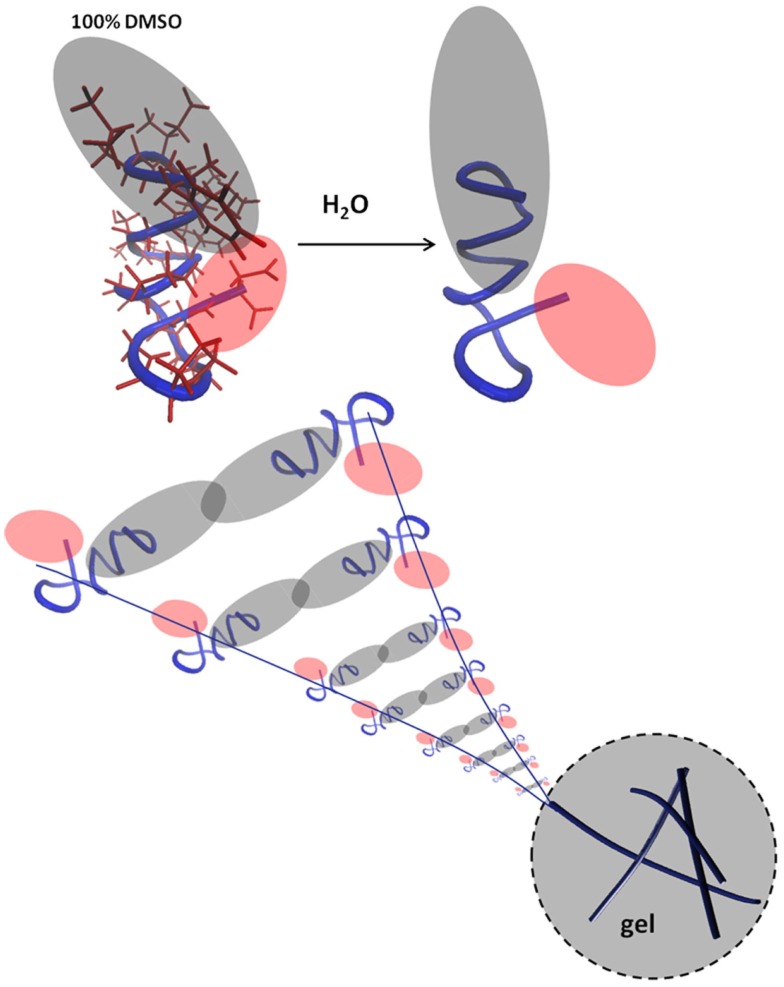
**The peptide interactions that form a fibril**. Reprinted with permission from Ref. (98). Copyright 2012 Biophysical Society.

h9e is a peptide hydrogel initially designed for drug delivery of a H1N1 vaccine (94) (Figure 3). It was designed by rationally combining functional native domains from the spider flagelliform silk protein and the trans-membrane segment of human muscle l-type calcium channel, resulting in a peptide with the sequence FLIVIGSIIGPGGDGPGGD (95, 96). h9e can produce a viable matrix for 3D encapsulation of MCF-7 cells (97), and has the unique property of displaying shear flow only when gelation is induced in the presence of calcium but not when calcium is absent. Calcium also increases the speed of gelation (98). To produce a matrix for cell encapsulation, the lyophilized peptide is dissolved into sodium bicarbonate and then added to cells in culture medium. Within 15 min, the mixture can solidify into a gel with a final stiffness of 500 Pa (97). The gel has no apparent negative effect on cell viability but cells divide slower as compared to 2D, an effect not uncommon in 3D culture constructs. Interestingly, 3D encapsulated cells displayed increased sensitivity to cisplatin compared to 2D cultures and the matrix is suitable for a variety of analytical methods including Western blotting, fluorescence microscopy, and trypan blue staining upon isolating the cells from the gel.

### FEFK and FEFKEFK

**Figure 4:**
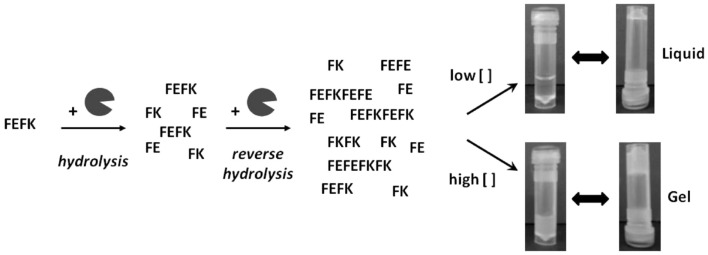
**Interaction between FEFK and enzyme**. Reprinted with permission from Ref. (102). Copyright 2013 American Chemical Society.

FEFK and FEFKEFK form a hydrogel upon a unique enzymatic interaction with a metalloproteinase (99) (Figure 4). FEFK is a short chain peptide that does not form a gel on its own but in the presence of the metalloproteinase thermolysine, the peptide is broken down and rebuilt into longer chains that do gel (100). This is a different gel strategy compared to most peptide gels which depend on pH, ionic salts, or light (101). The final makeup of the gel is determined by the initial concentration of FEFK, and the mechanical properties are determined by the initial enzyme concentration (102). Gelation can also be controlled by the temperature used to initiate the reaction (103, 104). To prepare the cell–gel constructs, FEFK is dissolved in PBS, loaded into a syringe, and the enzyme is added. After incubation for 5 min, cells can be added and the solution is injected in a well, requiring frequent media change in the initial 1 h of incubation to remove the enzyme. The gel takes about 10 min for gelation and finishes around 2000 Pa (105, 106). The hydrogel construct has been used for successful culture of fibroblasts and osteoblasts and no negative effects of the enzyme used for gelation were observed although about 40% of the enzyme remained within the gel at day 7 (105). Multiple cell viability studies report biocompatibility of the gel but the most in depth biological investigation was on the ability of osteoblasts to mineralize the gel showing increased calcium phosphate deposits and an increase in gel stiffness as the cells deposit calcium and extracellular proteins, indicating bone formation (106). The cell–hydrogel constructs can be used for live dead staining and isolation of cells from the gel. Additional studies have shown that the peptide can be functionalized with polymers without affecting the gel (107).

### K(SL)3RG(SL)3KGRGDS

K(SL)3RG(SL)3KGRGDS is an amphiphilic peptide with a non-polar middle surrounded by charged side regions, called a multi domain peptide (108). It forms beta sheets in aqueous solution by dimerizing to protect the non-polar core (109). The fibers had a diameter of 6 nm, expressed RGD for cell binding, and were susceptible to cleavage by cells (110). To form the cell–gel construct, the peptide was dissolved in water with sucrose and cells in PBS were added to the peptide solution starting the gelling process. To visualize cells, samples can be embedded and microtomed before being processed for microscopy (111). Cell viability assays revealed that addition of the RGD sequence slightly improved viability, but interestingly the viability was much more influenced by the location of the serines within the peptide sequence. In addition, incorporation of growth factors into the gel to improve culture conditions showed that addition of FGF improved the proliferation of fibroblasts but TGF-β had the opposite effect (111). While studies in cancer cells are still lacking, using such a construct dental pulp stem cells were successfully cultured and the cells were able to break down the gel and build up their own ECM, demonstrating the capability to create an engineered dental pulp (111). Using this peptide as base, studies were performed to see how changing the non-polar core to amphiphilic rings would affect gelation. While the basic nanofibrous morphology was retained in all cases, particular core residues resulted in switching from antiparallel hydrogen bonding to parallel bonding. In some cases, this resulted in more brittle fibers but fibrils always formed (112). When the serines in the serine leucine repeats were changed to threonine, the hydrogel still formed and encapsulated cells proliferated and broke down the scaffold, but the cells on the threonine scaffold grew much slower, especially if RGD was missing (113).

### MAX1

**Figure 5:**
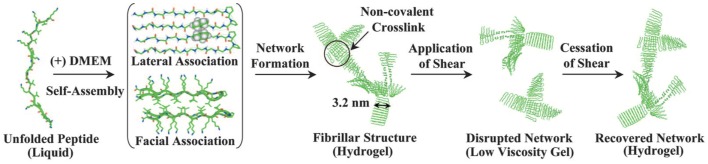
**MAX1 and MAX8 fibril formation**. Reprinted with permission from Ref. (114). Copyright 2007 National Academy of Sciences.

MAX1 is an amphiphilic peptide with the sequence VKVKVKVK-VDPPT-KVEVKVKV (54) (Figure 5). Gelation is triggered by a combination of salt concentration, pH, and temperature (114). These factors lead to charge screening which causes the peptide to fold into a beta hairpin, the hairpins then associate into fibrils forming the network through physical bonds (115). MAX1 gelation takes approximately 30 min to complete, which results in a heterogeneous cell distribution because the cells are able to sink through the gel. In order to ensure a homogenous cell distribution MAX8 was created by substituting a glutamic acid for a lysine, speeding up the gelation time to 1 min (114). To form a cell–gel construct, cells in serum-free medium are added to peptide dissolved in Hepes buffer and the construct is allowed to gelate before serum-containing medium is added to the culture. Using such a construct, mesenchymal stem cells, DAOY, Panc-1, and MG63 cells have been successfully cultured encapsulated within the gel (116, 117) (and our unpublished observations). The cell–hydrogel constructs can be assessed by fluorescence microscopy and MTT assays have been successfully performed. The fibrils have a 3.2 nm × 2 nm cross section (115) and the stiffness is around 1000 Pa, which can be controlled by changing the weight percent of the peptide and the speed of gelation which controls the number of branch points (118). MAX1 and MAX8 are shear thinning material allowing for gel injection while protecting its cargo (119). Thus, MAX8 can be used to deliver cells for therapy via syringe injecting while at the same time protecting the cells from shear forces (117). Furthermore, these hydrogels can be used for sustained delivery of active compounds including drugs. Studies have shown that encapsulated compounds may be protected from inactivation resulting in a consistent drug release over increased periods of time (119, 120) and *in vivo* biocompatibility has been demonstrated (116) (and our unpublished observations). The result is a highly customizable physical gel that shear thins and reheals. Gelation is triggered by physiological conditions allowing for easy culture setup without requiring the addition of harmful chemicals or organic reagents. It is mechanically robust with control over the storage modulus and a range of 500–10,000 Pa, thereby providing a very versatile 3D culture scaffold.

## Conclusion

The field of 3D cell culture is rapidly expanding due to new techniques and technologies that allow for this more complex culture system. It has resulted in new cell models that allow for the investigation of cellular phenotypes previously only seen *in vivo*. Much of the success is owed to the biocompatibility and bioactivity of natural materials and many current synthetic materials are designed by examining those found in nature. And while there is and will be a place for natural cell scaffolds in cellular investigation, it does not mean that the field needs to stop there. The advantages offered to cell culture by synthetic peptide scaffolds are great and will allow for in depth study of how cells interact with their environment and how the environment interacts with the cells. The biocompatibility and chemically defined nature of the materials results in a positive growth environment without the uncontrolled effects of unknown growth factors and proteins present. The synthetic nature of material synthesis allows for a consistent material without batch-to-batch variation, and it makes it simple to make variations on the scaffold as the researcher sees fit. All of these variables come together to give a cell culture system where the researcher has total control of the cellular environment and can trust that the results they are seeing are because of the changes they made. Having reliable, more *in vivo* like culture systems opens up a plethora of new possibilities in cancer research, from mimicking ECM environment to designing multi-cellular, tumor-like systems to developing culture systems that favor tumor stem cells. It is only a matter of time that the use of such complex platforms in drug discovery will result in novel lead compounds to support our quest to find a cure for the most aggressive and deadly cancers.

## Conflict of Interest Statement

The authors declare that the research was conducted in the absence of any commercial or financial relationships that could be construed as a potential conflict of interest.

## Abbreviations

2D, two-dimensional; 3D, three-dimensional; ECM, extracellular matrix; Fmoc-FF, fluorenylmethoxycarbonyl-diphenylalanine; Fmoc-RDG, fluorenylmethoxycarbonyl arginine–glycine–aspartic acid; HA, hyaluronic acid; PEG, polyethylene glycol.
